# The potential for political leadership in HIV/AIDS communication campaigns in Sub-Saharan Africa

**DOI:** 10.1080/16549716.2017.1270525

**Published:** 2017-02-03

**Authors:** Abraar Karan, Emily Hartford, Thomas J. Coates

**Affiliations:** ^a^ Department of Health Policy & Management, Harvard T.H. Chan School of Public Health, Boston, MA, USA; ^b^ Center for World Health, David Geffen School of Medicine, University of California Los Angeles, Los Angeles, CA, USA

**Keywords:** Political leadership, HIV/AIDS, Sub-Saharan Africa

## Abstract

**Background**: The HIV/AIDS epidemic has become a point of important political concern for governments especially in Sub-Saharan Africa. Clinical and public health interventions to curb the epidemic can be greatly enhanced with the strategic support of political leaders.

**Objective**: We analyzed the role of national political leadership in large-scale HIV/AIDS communications campaigns in 14 countries in Sub-Saharan Africa.

**Methods**: We primarily reviewed grey and white literature published from 2005–2014. We further triangulated data from in-person and phone interviews with key public health figures.

**Results**: A number of themes emerged supporting political leaders’ efforts toward HIV/AIDS program improvement, including direct involvement of public officials in campaign spearheading, the acknowledgment of personal relationship to the HIV epidemic, and public testing and disclosure of HIV status. Areas for future improvement were also identified, including the need for more directed messaging, increased transparency both nationally and internationally and the reduction of stigmatizing messaging from leaders.

**Conclusions**: The political system has a large role to play within the healthcare system, particularly for HIV/AIDS. This partnership between politics and the health must continue to strengthen and be leveraged to effect major change in behaviors and attitudes across Sub-Saharan Africa.

## Background

Political leadership is a critical pillar of a country’s healthcare system with great potential for impact. Political leaders have the opportunity not only to develop or improve the health system by drafting and passing bills, but also to use their inherent influence on the public to improve health attitudes and beliefs. Unfortunately, we have limited data within the public health field on how political leaders influence health outcomes.

The HIV epidemic is a prime example of the opportunity for political leadership to fundamentally redirect health attitudes and possibly, as a result, behaviors among a population. Since the beginning of the HIV epidemic, important factors relating to the growth of the epidemic have centered on people’s sexual choices (e.g. number of total lifetime partners, number of concurrent sexual partners, duration of time between new partners, age of sexual debut, use of condoms or other protective measures, type of sexual intercourse). These choices are influenced in large part by individual decision-making, societal norms and expectations, cultural context, and subsequent consequences including health outcomes, but also legal and economic repercussions. The political system, particularly through elected officials such as the President and Prime Minister, can also have an influence on people’s knowledge and attitudes with regard to HIV transmission. However, there is limited data on the way in which various countries have utilized their political systems to promote aspects of national public health responses to HIV.

Given that the HIV epidemic is heavily concentrated in Sub-Saharan Africa, a region with a history of political instability, it is especially important to study the role of political leadership within Sub-Saharan African countries. Case studies comparing various leadership decisions and ideologies in Sub-Saharan Africa have reflected the importance of high-level leadership, including that of the President, Vice President, First Lady, Ministers of Health, Prime Ministers, international Ambassadors, and other figures of political influence [1–3]. While it is essential to broadly acknowledge that political leadership is relevant to health outcomes, it is perhaps more useful to specify distinctive avenues through which politicians can impact healthcare. A study by Noar et al. focused on national HIV campaigns in Sub-Saharan Africa, North America, and Western Europe, many of which were started by country governments, and found that several campaigns have resulted in improvements in intention to change behavior and self-reported behavior change, as well as increased knowledge regarding HIV [4]. Still, data is limited regarding specific aspects of campaigns that have been successful or have failed.

Important areas of study in the field deserve specific attention, including direct involvement of public officials in spearheading health campaigns, the acknowledgment of a personal relationship to the HIV epidemic, public testing and disclosure of HIV status, voluntary circumcision by government officials, direct public health messaging with clear recommendations and objectives, and involvement of the country’s political leadership in international HIV/AIDS organizations.

## Methods

This was a qualitative study to analyze leadership practices as they relate to the HIV/AIDS epidemic in the following 14 countries: Malawi, Tanzania, Namibia, Zimbabwe, Zambia, Botswana, Uganda, Ethiopia, Rwanda, Kenya, South Africa, Mozambique, Swaziland, and Angola. Stratified sampling was utilized in the selection of countries to include in this study. Results represent a comprehensive range of experiences from those countries in the region with the highest HIV prevalence rates. Data was collected from June 2013 through April 2015. Data sources were included from 2005 through 2014 to represent a 10-year window.

We searched PubMed and Google with a number of keywords, including ‘HIV’, ‘AIDS’, ‘Sub-Saharan Africa’, ‘national campaigns’, ‘leadership’, ‘health politics’, and ‘communications’. We also gathered a large body of grey literature, including press releases and technical reports from country government agency websites and independent evaluating bodies. Due to the majority of our data being informed from the grey literature, our study methodology was not amenable to the PRISMA (Preferred Reporting Items for Systematic Reviews and Meta-Analyses) checklist. Inclusion criteria were as follows: articles whose primary focus related to the keywords aforementioned; articles in online newspapers and news blogs that could be cross-verified with more than one other source, so as to ensure a level of objectivity; and if the article was published after 2005 and before 2015. Exclusion criteria included: articles that were unable to be cross-verified with more than one source. Sources that covered the same news story were not all included; instead, the source that appeared highest on a Google search was cited with other sources utilized to cross-verify legitimacy of the information. Government agency websites were chosen with the same criteria and methodology as news sites. AK conducted the primary search, and articles were cross-verified and discussed by both AK and EH.

Furthermore, we conducted phone and in-person interviews with key contacts such as officials from Ministries of Health, CDC (Centers for Disease Control and Prevention) and USAID country branches, and with implementing partners such as the Johns Hopkins University Center for Communication Programs, which further informed our data and supported our ultimate conclusions. We did not directly utilize interviews from individuals in our manuscript and thus did not require informed consent from participants. No identifying or personal health information was collected from interviewees. Interviewees were verbally informed as to the nature of the inquiry.

Triangulation of the various data sources was utilized as a primary means of establishing the validity of our findings.

We broadly reviewed the following aspects of national leadership: (1) level of public communication, (2) type of public communication, (3) strategy employed during public communication, (4) transparency, and (5) involvement in both national and international activities.

## Results

### Direct campaign spearheading and public involvement

Several country governments have been directly involved in the leadership behind HIV/AIDS educational campaigns. The involvement of these officials can serve to legitimize and strengthen public backing and acceptance of local and national health movements. In Malawi, the *Tasankha* (‘Our Choice’) campaign, aimed at reducing multiple concurrent partnerships, was initiated in July 2010 with a public crowd led by Principal Secretary for HIV/AIDS, Dr. Mary Shawa, as well as other local political leaders [5]. The campaign included posters and songs, and helped create camaraderie between politicians and the public.

In 2013, Malawian President Dr. Joyce Banda hosted the UNAIDS/Lancet Commission where she supported and encouraged a 19-year-old woman to speak about her experience living with HIV [6]. Dr. Banda highlighted that the government was not only aware, but also supportive, of citizens living with HIV/AIDS [6].

In Tanzania, as part of the launch of the TUNAJALI campaign in September 2007, the President and First Lady Kikwete participated in a public HIV/AIDS testing session [7]. Several other high-ranking political figures such as the Prime Minister, US Ambassador, and 100 members of parliament (MPs) were also tested [7]. They also gave speeches, many of which included inspirational messages. In 2013, President Kikwete announced that Tanzania had tested 18 million people since he was publicly tested [7,8].

In Namibia, the President and First Lady Pohamba were also the head of their country’s national campaign, ‘Be Strong, Get Tested’, in 2010 [9]. Similarly, in Mozambique, the former First Lady Guebuza launched the ‘Unite for Children, Unite Against AIDS Campaign’ in 2005, which reached 550,000 people within 3 years [10].

### Directed and technical public health messaging

Some political figures have extended their influence into more directed and specific public health messaging. In Namibia, President Pohamba used both patriotic, emotional messaging in combination with direct advice on what individuals must actually do:

The ‘Be Strong – Get Tested’ campaign was designed as a call to action for all of our men, in every region, in every town, village and community to make your HIV testing plan and get it done. Do this for yourself, your family and your nation. [11]

Some campaigns, such as Uganda’s, focused specifically on certain aspects of treatment. First Lady Museveni headed a prevention of mother to child transmission (PMTCT) campaign focusing on the Option B+ treatment guidelines. Moreover, she was direct in her speeches, telling couples to use Option B+ to prevent transmission to children [12].

In Rwanda, Dr. Anita Asiimwe, the Minister of Health for Public Health and Primary Health Care, spoke to a crowd on World AIDS Day 2011 on very focused topics, including issues of transactional sexual relations, the role of parents protecting their children, as well as abstinence, monogamy, and condom use [13]. Moreover, her speeches were focused on urging citizens to complete a voluntary counseling and testing (VCT) campaign started by the government 6 months earlier.

The Kenyan government also utilized direct messaging, but lacked participation of high-profile figures. Dr. Francis Kimani, the country’s Director of Medical Services, asked men to take a more involved role in supporting women and children to access testing and treatment services [14]. However, more identifiable figures such as sports or movie stars or higher political leadership, such as the President, First Lady, or Prime Minister, have not yet participated in such a campaign.

### Public testing and intervention usage by government officials

Some of the most successful efforts to bolster HIV communication campaigns have been through demonstrative events. Public testing of officials has been employed in several countries (Malawi, Tanzania, Zimbabwe, Zambia, Uganda, Ethiopia, and South Africa) by Presidents, Parliamentarians, Ambassadors, and other political figures. Zimbabwe led a particularly strong effort in 2012; Blessing Chebundo, an MP who runs the Parliamentarians Against AIDS group, initiated a circumcision campaign during which 10 MPs were circumcised and 150 more committed to doing so in the future [15]. In South Africa, President Zuma has attempted to reduce the immense stigma created out of former President Mbeki’s staunch AIDS denialism in the early 2000s; in 2010, President Zuma publicly revealed that his fourth HIV test was negative. He also, through the following message, began to create a new national mentality around HIV: ‘We have to make all South Africans understand that people living with HIV have not committed any crimes’ [16].

### Relation of familial experiences with HIV

A few African leaders have attempted to normalize the stigma associated with HIV by publicly acknowledging the impact of HIV in their personal lives. In 2004, ex-President of Malawi, Bakili Muluzi, was publicly tested and spoke openly about losing his brother to AIDS [17]. In Zimbabwe, long-time ruler President Robert Mugabe discussed losing several members of both his cabinet and family to HIV over the course of his presidential term [18]. Similarly, former Zambian President Kenneth Kaunda was publicly tested in 2002 and admitted to losing one of his sons to HIV/AIDS [19].

### International HIV/AIDS leadership

Participation in multinational organizations is another avenue via which African state heads have combated HIV. The wife of Ethiopia’s former Prime Minister, Ms. Azeb Mesfin, is the head of the Organization of First Ladies Against HIV/AIDS [20]. International leadership demonstrates commitment to HIV efforts globally, and often translates to local involvement as well. Of importance, Ms. Mesfin participated in a public testing event with the US Ambassador to Ethiopia Arelia Brazeal and State Minister Mulu Ketsala, and was instrumental in ensuring the AIDS Healthcare Foundation was one of the few organizations to be present at the 125th Addis Ababa city anniversary, which garnered significant media attention [21]. In Mozambique, the former First Lady Maria da Luz Guebuza was recently appointed the patron of the ‘Global Plan towards the elimination of new HIV infections among children by 2015 and keeping their mothers alive’ [22].

### Areas of existing opportunity

African government leaders have led very strong efforts to improve HIV communication programs and encourage their citizens to get tested and treated, as well as to reduce existing stigma. Still, we found there to be potential opportunities to improve public messaging.

Many African government officials have given speeches that used indirect public health messaging which can be confusing and counterproductive. In Zimbabwe, the message, ‘Be a winner, get circumcised’ was unclear, with people wondering what it is they would be ‘winning’, as communicated by the UNAIDS country director [23]. In Swaziland, the 2012 circumcision campaign ‘Circumcise and Conquer’ was a noted failure although it did receive substantial backing from King Mswati. Among other reasons, certain phrases such as ‘Lisoka lisoka negkusoka’, which translates into, ‘The lover boy is a lover boy because of circumcision’, were off-putting to married men [24].

In Zambia, the campaign messaging might benefit from more specific public health instruction. For instance, the following from President Kenneth Kaunda used motivational language but the actual instruction was vague from a clinical and public health perspective:

We support because we believe it is the right thing to do. The best support should come from the heart, out of conviction that you are doing the right thing. We all need to do what we can to fight this scourge. … No amount of effort is too small, everyone should just do what they can do and together we can achieve. You will find us here. Whatever we can do, we will be there. [25]

Similarly, in Botswana, President Khama delivered the following speech at World AIDS Day in 2011 in relation to the *O Icheke* campaign focused on reducing concurrent partnerships:

My message to you is very basic – prevention starts with you, today; prevention is your responsibility; prevention is not just a choice; prevention is the choice. But our prevention efforts must deliver greatest returns to investment. We must focus our efforts where they are most needed and can make an impact. [26]

More specific messages about different types of prevention, how effective they are, and when to use them in addition to general messages typically delivered by leadership could be useful when commanding the attention of the country.

Moreover, some countries had particularly poor international visibility with regards to the involvement of public leaders in international health and HIV organizations, and were notably absent from our sources of information including both white and grey papers on the Internet from news sources, government websites, and other forums. We did not find much information from Zambia, Botswana, and Angola in particular. In Zambia, the current President Sata has been involved with delivering updates on country-level data, but there is very little media coverage indicating that he has been involved in campaign spearheading. Additionally, the *O Icheke* campaign in Botswana was said to have had strong governmental backing and support, yet it was extremely difficult to find any coverage regarding what the government and its actors were actually doing to promote or launch the campaign itself. Recently, one of Botswana’s Ministers made a call to youth that ‘discipline and behavior change are key in defeating HIV/AIDS’ [27]. However, as mentioned previously, non-specific messaging such as this is not particularly useful. Angola had very little available recent data on public campaigns for HIV/AIDS. The Angolan Network of HIV/AIDS Organizations has been urging the national government to launch a countrywide campaign [28]. Some campaigns in the country have included one in 2012 by a private oil firm partnering with the National Institute of Fight Against HIV/AIDS and another in 2005 focusing on education in schools but lacking in any major political leadership presence [29,30]. Interestingly, Zambia, Botswana, and Angola have very different HIV prevalence rates. In 2013, Angola had a prevalence of just 2.4%, whereas Zambia was much higher at 12.5%, and Botswana one of the highest in the world at 21.9% [31].

Additionally, several governments in Sub-Saharan Africa have been involved in stigmatizing behavior and false health messaging. In Uganda, President Museveni has denounced male circumcision in the past as a strategy that will make Ugandans more complacent and promiscuous [32]. In Swaziland, an MP called for iron branding on the buttocks of people living with HIV/AIDS (PLWHA) [33]. Moreover, when South African President Zuma mistakenly claimed that he showered to ‘minimize the risk of contracting HIV’, he created confusion and misunderstanding regarding both treatment and prevention of HIV [34]. Statements such as these create fear and challenge the uptake of large-scale governmental HIV programs in the future.

## Discussion

Political leadership as it relates to the HIV epidemic in Sub-Saharan Africa has been strong on several fronts, although there are areas of opportunity for improvement as well. The relationship between political stability and transparency as it relates to health outcomes has been studied previously, with quite promising avenues for future development. For example, the stability and transparency of governments in emerging economies have been shown to be strongly associated with increased ART (antiretroviral therapy) provision [35]. Yet, other studies have elucidated the complexity of the commitment of political institutions within epidemic countries – findings suggest that elected leaders are no more committed than those who are autocratic; that freedom of the press is highly linked to political responsiveness; and that a country’s economic inequality is negatively associated with leadership attention toward HIV/AIDS [1]. The current shift toward increased country ownership of HIV/AIDS programs, as outlined by Collins and Beyrer, will be highly dependent on the integrity and proactivity of national leadership [36]. More research is needed to better understand the way that complex political structures interact with the health sphere, and how these relationships can help advise country leaders toward more effective public health responses.

We found that in several countries, including Tanzania and Namibia, leading public officials as essential as the Presidents and First Ladies have had direct involvement in launching campaigns for voluntary HIV counseling and testing, circumcision, and prevention. Some of these leaders have personally been tested as exemplars, and others have made commitments to be circumcised so as to set a public precedent. In many instances, leaders spoke openly about cabinet members and even family members who had succumbed to HIV, a move that began to de-stigmatize PLWHA and frame the problem as one that everyone, including the President, had to confront. Furthermore, there is a developing body of African leaders who have collaborated to make the battle against HIV one of international and pan-African concern. In both Ethiopia and Mozambique, the First Ladies have demonstrated international leadership in HIV politics, and this has set the stage for sharing of knowledge including successes and failures. Still, despite Africa’s strong political response to the epidemic, we have also highlighted areas for improvement, including a stronger oversight of national messaging that can otherwise be interpreted as stigmatizing or ambiguous. Approaches that disregard scientifically sound interventions, such as regarding circumcision, are bound to obstruct progress and uptake of preventative solutions. Moreover, most countries need to increase both national and international transparency, and aim to popularize their campaigns with the local populace as well as neighboring countries. Also, in many countries, we found that only lower-level leadership was significantly involved in the healthcare sector. It is critical that all heads of state make it a priority to become involved in the national public health response to the HIV epidemic.

Increasing effective Sub-Saharan African government involvement in the HIV epidemic will have to be encouraged and advised by civil societies, the health sector, and the international community. Governmental and non-governmental organizations, ministries of health, academic institutions, and hospitals must collectively leverage their voice to advocate for increased government leadership. Political leaders need to work more closely with their health advisors as well, particularly when crafting messaging for national health campaigns. The input of civil society will be particularly useful in creating effective campaigns. Moreover, the international community, through multinational and intergovernmental organizations such as the United Nations, World Health Organization, and the South African Development Community, must provide input for the sufficient involvement of political leaders in the healthcare sector generally and for HIV specifically. A set of standards should be developed for what constitutes a sufficient level of political involvement beyond the usual fiscal measures such as percentage of GDP allocated toward the healthcare sector. The international community should support country leaders in monitoring the HIV epidemic, actively looking at new social and biomedical solutions, and supporting their effective roll-out.

By highlighting instances of strong political commitment to curbing the HIV epidemic, we acknowledge that African leaders have been active on the political front. However, we feel that substantially more work needs to be done on this front. Through further data collection, we encourage the creation of a standardized scale that can be utilized to quantitatively and qualitatively assess the level of political leadership a country’s government has displayed with regards to HIV and other health conditions. Reference to Bor’s previous work on this through utility of the AIDS Program Effort Index (API) is an important contributor to this growing field of health politics and should be studied as well [1]. A universal ‘political commitment to health’ scale would ideally be used by country governments to self-assess and compare their level of political commitment with that of other governments. It would also be amendable for use by the international community and civil society to encourage accountability.

The difficulties of assessing leadership and its relevance to health outcomes are manifold. One important point to note is that many political leaders don’t command the respect and popular support of their citizenry, which means that ‘strong’ leadership does not necessarily translate into behavior change or improved health outcomes.

## Conclusions

Political leadership as it relates to healthcare has yet to be systematically studied or measured in depth. The HIV/AIDS epidemic in Sub-Saharan Africa demands strong political willpower from country governments to help shape health campaigns and influence the public’s attitude, knowledge, and practice with regards to curbing the epidemic. We present key themes and outline strong and weak practices that various sub-Saharan African governments have demonstrated in their responses to the HIV epidemic. We suggest the creation of an HIV/AIDS political leadership scale that can be utilized to systematically assess political commitment to HIV and the outcomes that are thereby affected. By having a tool by which to compare various governments in their commitment to battling HIV/AIDS, civil societies and the international community can demand more from political leaders in curtailing the spread of the illness.

## Paper context

We critically examined the specific leadership practices of the political elite in Sub-Saharan Africa as they related to the HIV/AIDS epidemic, particularly around communications campaigns such as speeches and rallies. We found that practices varied greatly across countries and had far-reaching externalities, including complex effects from unclear messaging and stigmatization. There needs to be further cross-national dialogue and analysis to emulate best practices and avoid harmful leadership tactics that are applicable globally.

## References

[CIT0001] Bor J. (2007). The political economy of AIDS leadership in developing countries: an exploratory analysis. Soc Sci Med.

[CIT0002] Parkhurst JO, Lush L (2004). The political environment of HIV: lessons from a comparison of Uganda and South Africa. Soc Sci Med.

[CIT0003] Allen T, Heald S (2004). HIV/AIDS policy in Africa: what has worked in Uganda and what has failed in Botswana?. J Int Dev.

[CIT0004] Noar SM, Palmgreen P, Chabot M (2009). A 10-year systematic review of HIV/AIDS mass communication campaigns: have we made progress?. J Health Commun.

[CIT0005] Johns Hopkins Center for Communication Programs (2010). Malawi BRIDGE II project launches “Tasankha” (our choice) mass media campaign. http://ccp.jhu.edu/malawi-bridge-ii-project-launches-tasankha-choice-mass-media-campaign/.

[CIT0006] Herald News (2013). Malawi’s president launches HIV/AIDS summit. http://www.heraldnews.com/article/20130630/Blogs/306309905.

[CIT0007] PEPFAR (2007). Tanzania launches national HIV/AIDS testing campaign. http://www.pepfar.gov/press/92135.htm.

[CIT0008] Daily News Tanzania (2013). Kikwete outlines Tanzania’s successes in HIV/AIDS fight. http://dailynews.co.tz/archive/index.php/local-news/18912-kikwete-outlines-tanzania-s-successes-in-hiv-aids-fight.

[CIT0009] IntraHealth (2010). “Be strong- get tested” campaign launched in Namibia. http://www.intrahealth.org/page/be-strongget-tested-campaign-launched-in-namibia.

[CIT0010] UNICEF (2016). Mozambique unites against AIDS. http://www.unicef.org/mozambique/media_2195.html.

[CIT0011] UNICEF (2011). A national campaign aims to increase Namibian men’s involvement in HIV health programmes. http://www.unicef.org/infobycountry/namibia_58044.html.

[CIT0012] Johns Hopkins Center for Communication Programs (2013). Uganda campaign seeks to end mother-to-child transmission. http://ccp.jhu.edu/towards-an-hiv-free-generation-uganda-launches-a-campaign-to-eliminate-mother-to-child-transmission-of-hiv/.

[CIT0013] Rwanda Biomedical Center (2011). Launching the WAD campaign in Bugesera district. http://www.rbc.gov.rw/spip.php?article145.

[CIT0014] UNAIDS (2012). Kenya launches campaign to stop new HIV infections among children by 2015 and keep their mothers alive. http://www.unaids.org/en/resources/presscentre/featurestories/2012/november/20121128kenyapmtct.

[CIT0015] DailyMail (2012). Nearly 70 Zimbabwean MPs to undergo circumcision as part of huge anti-HIV drive. http://www.dailymail.co.uk/news/article-2163195/Nearly-70-Zimbabwean-MPs-undergo-circumcision-huge-anti-HIV-drive.html.

[CIT0016] Times Live (2010). Red letter day as President Zuma discloses his HIV status. http://blogs.timeslive.co.za/hiv/2010/04/red-letter-day-as-president-zuma-discloses-his-hiv-status/.

[CIT0017] BBC News (2004). Africa leaders still shy about AIDS. http://news.bbc.co.uk/2/hi/africa/3482157.stm.

[CIT0018] The Zimbabwean (2012). Mugabe admits losing cabinet to HIV and AIDS. http://www.thezimbabwean.co/2012/03/mugabe-admits-losing-cabinet-to/.

[CIT0019] IRIN News (2010). Africa leaders go public with the test. http://www.irinnews.org/printreport.aspx?reportid=89005.

[CIT0020] UNAIDS (2011). First lady of Ethiopia commits to elimination of new HIV infections among children. http://www.unaids.org/en/resources/presscentre/featurestories/2011/december/20111207firstladies.

[CIT0021] AIDS Healthcare Foundation (2013). Treatment, testing, and prevention on the horn of Africa. http://www.aidshealth.org/archives/15808.

[CIT0022] UNAIDS (2013). First lady of Mozambique appointed as patron of the global plan. http://www.unaids.org/en/resources/presscentre/featurestories/2013/july/20130731mozambique.

[CIT0023] All Africa (2013). Zimbabwe: resistance hinders circumcision program. http://allafrica.com/stories/201305200210.html.

[CIT0024] PBS (2012). Why a US circumcision push failed in Swaziland. http://www.pbs.org/newshour/updates/health-july-dec12-swaziaids_07-05/.

[CIT0025] UNICEF (2011). “Brothers for life” media campaign to raise HIV awareness in Zambia. http://www.unicef.org/media/media_59821.html.

[CIT0026] Republic of Botswana (2011). His excellency president Lieutenant General Seretse Khama Ian Khama world AIDS day commemoration. http://www.gov.bw/Global/NACA%20Ministry/wana/World%20AIDS%20Day%202011%20%2023%20Nov%20%202011%20By%20HE.pdf.

[CIT0027] Leadership Newspaper (2016). Minister urges youths in Botswana to prevent new HIV infections. http://leadership.ng/news/519639/minister-urges-youths-botswana-prevent-new-hiv-infections.

[CIT0028] Panapress (2011). Official wants Angola to step up HIV/AIDS awareness campaign. http://www.panapress.com/Official-wants-Angola-to-step-up-HiV-AiDS-awareness-campaigns—12-756536-66-lang2-index.html.

[CIT0029] UNICEF (2005). HIV/AIDS campaign in Angolan schools. http://www.unicef.org/media/media_28178.html.

[CIT0030] All Africa (2012). Angola: HIV/AIDS awareness campaign launched. http://allafrica.com/stories/201210190377.html.

[CIT0031] World Bank (2015). Prevalence of HIV, total (ages 15–49). http://data.worldbank.org/indicator/SH.DYN.AIDS.ZS.

[CIT0032] The Africa Report (2014). HIV/AIDS: put padlocks on your private parts – Museveni. http://www.theafricareport.com/East-Horn-Africa/hivaids-put-padlocks-on-your-private-parts-museveni.html.

[CIT0033] IOL News (2009). Brand HIV-positive people on the buttocks. http://www.iol.co.za/news/south-africa/brand-hiv-positive-people-on-the-buttocks-1.444283#.VSwxnEK96Qx.

[CIT0034] BBC (2006). SA’s Zuma ‘showered to avoid HIV’. http://news.bbc.co.uk/2/hi/africa/4879822.stm.

[CIT0035] Young Nicola Man W, Worth H, Kelly A (2014). Is endemic political corruption hampering provision of ART and PMTCT in developing countries?. J Int AIDS Soc.

[CIT0036] Collins C, Beyrer C (2013). Country ownership and the turning point for HIV/AIDS. Lancet Glob Health.

